# Diagnostic Accuracy of Vitreous Cytology in Patients with Vitreoretinal Lymphoma

**DOI:** 10.3390/jcm11216450

**Published:** 2022-10-31

**Authors:** Donghyun Lee, Junwon Lee, Ji-Hae Nahm, Min Kim

**Affiliations:** 1 Department of Ophthalmology, Inha University Hospital, Inha University College of Medicine, Incheon 22332, Korea; 2 Department of Ophthalmology, Gangnam Severance Hospital, Yonsei University College of Medicine, Seoul 03722, Korea; 3 Department of Pathology, Gangnam Severance Hospital, Yonsei University College of Medicine, Seoul 03722, Korea

**Keywords:** eye neoplasm, masquerade syndrome, vitreous cytology, lymphoma

## Abstract

(1) Background: To determine the diagnostic value of vitreous cytology in patients with vitreoretinal lymphoma (VRL) and evaluate its diagnostic accuracy relative to that of other diagnostic tests. (2) Methods: In total, 38 eyes from 38 patients with VRL who underwent diagnostic vitrectomy and were followed up for at least 6 months were analyzed. The clinical manifestations and VRL diagnostic rates for all diagnostic tests were determined. (3) Results: The presence of vitreous cells/opacity was the most common ophthalmic finding (97.4%), followed by sub-retinal pigment epithelial infiltration (65.8%) and retinal hemorrhage (21.1%). The VRL diagnostic rates were 89.3% for interleukin (IL)-10 levels > 50 pg/mL; 82.1% for IL-10/IL-6 ratios > 1; 60.0% and 63.3% for immunoglobulin heavy chain and kappa light chain clonality assays, respectively; and 44.4% for vitreous cytology. The VRL diagnostic rate for vitreous cytology was significantly lower in the steroid pretreatment group than in the non-steroid pretreatment group (*p* = 0.007). (4) Conclusions: The VRL detection rate for vitreous cytology was lower than that for the other tests, especially in patients who received steroid pretreatment. These findings suggest that even if vitreous cytology findings are negative, other tests and characteristic fundus findings should be evaluated to confirm VRL diagnosis.

## 1. Introduction

Vitreoretinal lymphoma (VRL) is an intraocular malignancy that may present in a manner consistent with that of severe intermediate or posterior uveitis [1,2]. Histologically, most cases of VRL involve high-grade B-cell lymphomas [3]. Because VRL often involves the central nervous system, early diagnosis and active treatment are required. VRL has an incidence of approximately 0.047 per 100,000 individuals [4] and occurs in adults aged 30–80 years [5,6,7]; immunosuppression is a risk factor for VRL [8,9].

The most reliable diagnostic method for VRL is vitreous cytology. The typical histological features of lymphoma cells are a prominent nucleus, a coarse chromatin pattern, and a relative lack of cytoplasm [10,11]. However, the number of vitreous samples that can be obtained from vitreous tapping is limited. Moreover, even when diagnostic vitrectomy is performed, VRL remains difficult because of the risk of tumor cell loss or direct insult to the tumor cells by the vitrectomy cutter [11,12]. To compensate for these shortcomings, alternative methods have been introduced; these include measurement of the interleukin (IL)-6 and IL-10 levels in the vitreous cavity [13], immunoglobulin gene rearrangements for clonality assessment [14], and detection of oncogenic myeloid differentiation primary response gene 88 (*MYD88*) mutations [15].

Nevertheless, when steroids are administered for treating posterior uveitis in patients with suspected VRL, lymphoma cell lysis may occur and cause difficulties in establishing an accurate diagnosis. In such cases, it is recommended that practitioners maintain a waiting period between steroid treatment initiation and vitreous infiltration induction and subsequent diagnostic vitrectomy [16]. However, this approach is associated with delayed diagnosis and treatment. In this study, we aimed to compare the sensitivities of various diagnostic tests used for confirming VRL and propose an alternative to vitreous cytology in patients undergoing steroid pretreatment for posterior uveitis.

## 2. Materials and Methods

This study was conducted in accordance with the tenets of the Declaration of Helsinki and was approved by the institutional review board of Gangnam Severance Hospital (approval number: 3-2021-0006); the review board waived the requirement for informed consent.

### 2.1. Study Design and Inclusion and Exclusion Criteria

This was a retrospective, observational study that was based on the medical records of patients who visited our tertiary medical institutions between May 2005 and August 2021. Patients were eligible for this study if they had VRL, underwent diagnostic vitrectomy for vitreous cytology, and were followed up for at least 6 months. The diagnostic criteria for VRL were positive vitreous cytology findings or characteristic funduscopic features/vitreous opacity with a favorable response to intravitreal (IVit) methotrexate (MTX) treatment. Patients were excluded from the study if they had another ophthalmic disease affecting their vision, such as diabetic retinopathy, age-related macular degeneration, or glaucoma, among others.

### 2.2. Diagnostic Procedures and Statistical Methods

Data on the patients’ demographic, medico-surgical, and treatment characteristics were extracted. Data on the corrected visual acuity, intraocular pressure, slit lamp findings, funduscopic examination findings, and findings of various imaging tests were also collected. All patients underwent a 23-gauge or 25-gauge three-port pars plana vitrectomy at a low cutting rate of 500–1000 cuts/min. Initially, with the infusion stopped, 1–2 mL of the undiluted vitreous sample was collected; this was followed by the collection of the diluted vitreous sample. The undiluted vitreous sample was subjected to vitreous cytology and tests for determining the IL-6 and IL-10 levels. The diluted vitreous sample was subjected to immunoglobulin heavy chain (IGH) and immunoglobulin kappa light chain (IGK) clonality assays, bacterial and fungal staining and culture, and other tests specific to potential causative diseases as required. Vitreous cytology confirmed whether the number of cells in the sample was sufficient for diagnosis and whether lymphoma-specific cells (with their prominent nuclei, coarse chromatin patterns, and a relative lack of cytoplasm) were visible; cellular paucity was described as an insufficient number of cells in the sample. Furthermore, findings from biopsies of other lymphoma-affected organs were examined if available. SPSS version 25.0 (IBM Inc., Armonk, NY, USA) was used for all statistical analyses. Descriptive statistics are reported; a subgroup analysis was performed using the Chi-square or Fisher’s exact test to determine the effect of steroid pretreatment on diagnostic accuracy.

## 3. Results

A total of 38 patients were included in this study. Among these, 27 (71.1%) patients presented with bilateral VRL during the follow-up period. The mean age of the patients was 62.5 ± 11.9 years, and 63.2% (*n* = 24) of the patients were women. Furthermore, 10 (35.7%) and 8 (21.1%) patients had hypertension and diabetes, respectively. There were no immunocompromised patients. Thirty-four (89.5%) patients received IVit MTX; the average number of injections was 14.7 ± 6.9. Twelve patients with primary VRL exhibited central nervous system involvement during the follow-up period, and the treatment patterns were as follows: 90.9% of the patients received IVit MTX combined with systemic chemotherapy, while 40.9% of the patients received IVit MTX combined with systemic chemotherapy and regional radiation therapy. Patients were followed up for an average of 36.3 ± 33.7 months, and 28.9% (*n* = 11) of the patients died of the disease during the follow-up period (Table 1).

### 3.1. Ocular Findings at Diagnosis

**Figure 1:**
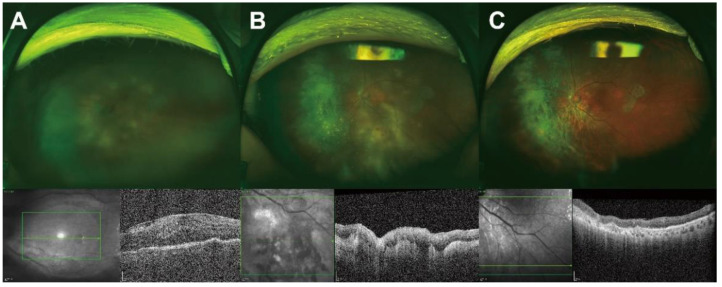
Representative ocular findings before and after diagnostic vitrectomy. A 75-year-old woman visited the outpatient clinic with a complaint of decreased visual acuity in her left eye that began 3 months prior to presentation. She was receiving oral prednisolone for chronic uveitis. At the first examination, the visual acuity was 20/200 and the intraocular pressure was 11 mmHg. Slit-lamp examination revealed inflammatory cells in the anterior chamber and vitreous cavity. (**A**) Funduscopic examination revealing vitreous opacity with multifocal, subretinal, yellowish granular infiltration. Optical coherence tomography (OCT) images showing the epiretinal membrane, dense infiltration of the sub-retinal pigment epithelium (subRPE), lumpy-bumpy choroid, subretinal fluid, and intraretinal fluid. Following these findings, a diagnostic vitrectomy was performed. (**B**) After diagnostic vitrectomy, a yellowish subretinal infiltration was clearly visible, and subRPE infiltration and lumpy-bumpy choroid were also visible on OCT images. Accurate diagnosis with vitreous cytology was not possible due to cellular paucity; however, a diagnosis of vitreoretinal lymphoma was confirmed through an IGK gene clonality assay. (**C**) The lesions improved after four injections of intravitreal methotrexate.

The mean initial best-corrected visual acuity was 0.8 ± 0.9 logMAR (0.2 ± 0.1; Snellen equivalent visual acuity), and the mean intraocular pressure was 13.4 ± 3.9 mmHg. Inflammatory cells were observed in the anterior chamber in 44.7% of the patients (grade: 1.4 ± 1.3 (Standardization of Uveitis Nomenclature)). Vitreous cells or haziness were observed in 97.4% of patients, whereas retinal pigment epithelium infiltration and retinal hemorrhage were observed in 65.8% (*n* = 25) and 21.1% (*n* = 8) of patients, respectively (Figure 1, Table 2).

### 3.2. Origin and Involvement Patterns of Vitreoretinal Lymphomas

Overall, a histological diagnosis of diffuse large B-cell lymphoma was confirmed in 57.9% of the VRL cases; in the remaining 42.1% of the cases, a histological diagnosis was not possible. Primary VRL originating from the eye and primary central nervous system lymphoma originating from the brain accounted for 73.7% (*n* = 28) and 26.3% (*n* = 10) of the cases, respectively. The rate of brain involvement in cases of primary VRL during the follow-up period was 42.9% (*n* = 12; 17.9 ± 12.3 months). Central nervous system involvement was identified by magnetic resonance imaging during routine checkups in 75.0% (*n* = 0) of the patients or after the detection of newly developed neurological symptoms and visual field defects in 16.7% (*n* = 2) and 8.3% (*n* = 1) of the patients, respectively (Table 3).

### 3.3. Comparing the Diagnostic Values of Tests

The diagnostic accuracies of the confirmatory tests for VRL were compared. For vitreous cytology, the VRL detection rate was 44.4% (*n* = 12); cellular paucity was observed in 28.9% (*n* = 11) of the cases. IL analysis was performed in 73.7% of the patients; the detection rate of an IL-10/IL-6 ratio of >1 was 82.1% (*n* = 23). The detection rate of an IL-10 level of >50 pg/mL was 89.3% (*n* = 25). In the 78.9% of the patients who underwent IGH/IGK gene clonality assays, the detection rates for IGH and IGK positivity were 60.0% (*n* = 18) and 63.3% (*n* = 19), respectively. The detection rate for either the IGH or IGK gene clonality assay was 83.3% (*n* = 25; Table 4).

### 3.4. Effect of Steroid Pretreatment on Diagnostic Accuracy

Additional analyses were performed to determine whether the use of steroids before diagnostic vitrectomy affected the diagnostic test findings. A total of 31.6% (*n* = 12) of the patients received steroids before diagnostic vitrectomy. After excluding samples that could not be evaluated accurately due to cellular paucity, the rate of positive vitreous cytology findings in the steroid pretreatment group was 0.0% (*n* = 0); this was lower than the corresponding rate of 57.1% (*n* = 12) in the non-steroid pretreatment group (*p* = 0.020). Conversely, no significant differences were observed in the findings of the IL analysis or the IGH/IGK assays between the two groups (Table 5).

## 4. Discussion

In the present study, primary VRL was more common than VRL originating from the central nervous system or other organs. In addition, most patients (42.9%) with primary VRL exhibited brain involvement during follow-up. The VRL diagnostic rate for IL analysis was the highest, followed by that for the IGH/IGK gene clonality assays; the VRL diagnostic rate for vitreous cytology was the lowest, particularly in the steroid pretreatment group.

According to a recently published study, the most important tests for the diagnosis of VRL are diagnostic vitrectomy, an IL-10/IL-6 ratio > 1, positivity for *MYD88* gene mutations, and monoclonality [17]. In our study, IL analysis and IGH/IGK gene clonality assays were effective for VRL diagnosis; however, diagnostic vitrectomy had a relatively low diagnostic value due to steroid pretreatment and insufficiency in the number of samples withdrawn for analysis.

Identifying lymphoma cells in a vitreous specimen is key to the diagnosis of VRL. However, the diagnostic rate of VRL for vitreous cytology is 45–55% [11,18]. The reasons for such low detection rates may include the following: (1) lymphoma cells may not be detected in the samples obtained through vitreous tapping or diagnostic vitrectomy and (2) the vitreous specimens may be contaminated by other cellular structures, such as reactive T lymphocytes, necrotic cells, debris, and fibrin [7]. In this study, lymphoma cells were identified in only 44.4% of the patients diagnosed with VRL. Moreover, pathological diagnosis was impossible in 28.9% of the patients due to cellular paucity; these findings suggest the limitations of vitreous cytology under conditions wherein the detection of lymphoma cells may be difficult. Therefore, although vitreous cytology is the first-line diagnostic method for confirming VRL, a negative finding cannot definitively exclude it.

Cytokine analysis can be used for VRL diagnosis; the amount of IL-10 derived from tumor cells can be measured and used to calculate the IL-10/IL-6 ratio. As a diagnostic biomarker for VRL, this ratio has a sensitivity of 81–92% and a specificity of approximately 100% [11,13,18,19,20,21,22]. IGH/IGK gene rearrangement in B-cell lymphomas is also an important diagnostic tool for VRL; it has a sensitivity of 46–96% and a specificity of 85–100% [11,18,19,20,21,22,23]. In this study, IL analysis and IGH/IGK gene clonality assays had a high VRL diagnostic rate of >80%; thus, our findings confirm their usefulness for VRL diagnosis. Corticosteroids are important in the treatment of uveitis; in fact, IVit injection of steroids is common in clinical practice [24]. However, up to 2.5% of the patients referred for uveitis treatment may exhibit neoplastic masquerade [25]; initiating corticosteroid therapy before obtaining an accurate diagnosis can worsen the causative disease. In older adults, when the baseline visual acuity has severely deteriorated and posterior segment involvement is severe, neoplastic masquerade should be ruled out. However, corticosteroid pretreatment increases the likelihood of negative vitreous cytology findings due to the lymphocytic effect of steroids and tumor cell lysis during vitreous biopsy [16]. According to Carbonell et al., the administration of systemic corticosteroids within 2 weeks before diagnostic vitrectomy is not recommended because it interferes with the diagnostic yield [17]. In this study, the diagnostic sensitivity of vitreous cytology was lower in the steroid pretreatment group than in the non-steroid pretreatment group. However, the results of the IL analysis and the IGH/IGK gene clonality assays did not differ significantly between the two groups, suggesting that a cytokine assay or a polymerase chain reaction analysis for the immunoglobulin gene sequence may be diagnostically sensitive even in samples of very small volumes.

This study has some limitations. First, it is retrospective in nature. Second, patients for whom VRL diagnoses were not confirmed histologically were also included in the study population; thus, patients with lymphomas other than B-cell lymphomas may also have been included. Third, the number of patients who received pretreatment with steroids was relatively small. Finally, *MYD88* gene mutation test results were not analyzed in the study. Nevertheless, this study is meaningful because it reveals the diagnostic accuracy of IL analysis and the IGH/IGK gene clonality assays for VRL.

## 5. Conclusions

The present study suggests that vitreous cytology findings should be interpreted carefully in patients who have received steroid pretreatment because of high false-negative rates. In such patients, measuring the levels of IL-6 and IL-10 and immunoglobulin gene rearrangement for clonality assessments may help diagnose VRL.

## Figures and Tables

**Table 1:** Demographic characteristics of the study population.

	Count/Mean ± Standard Deviation
Patients (*n*)	38
Laterality (right/left)	17/21
Age (years)	62.5 ± 11.9
Sex (male/female)	14/24
Comorbidities	
Hypertension	10 (35.7%)
Diabetes mellitus	8 (21.1%)
Mean follow-up period (months)	36.3 ± 33.7
Treatment patterns for VRL	38 (100.0%)
IVit MTX (%, count)	34 (89.5%, 14.7 ± 6.9)
None	4 (10.5%)
Treatment patterns for CNS lymphoma	22 (57.9%)
IVit. MTX + Systemic CTx	20 (90.9%)
IVit. MTX + Systemic CTx + Brain/Eye RTx	9 (40.9%)
Death during follow-up	11 (28.9%)

VRL, vitreoretinal lymphoma; IVit, intravitreal; MTX, methotrexate; CNS: central nervous system; CTx, chemotherapy; RTx, radiation therapy.

**Table 2:** Ocular findings at diagnosis.

	Count/Mean ± Standard Deviation
LogMAR BCVA (Snellen equivalent)	0.8 ± 0.9 (0.2 ± 0.1)
IOP (mmHg)	13.4 ± 3.9
Anterior segment findings	
Keratic precipitates	2 (5.3%)
Corneal edema	1 (2.6%)
Cells/SUN grading	17 (44.7%)/1.4 ± 1.3
Posterior segment findings	
Vitreous cells or haziness	37 (97.4%)
SubRPE infiltration	25 (65.8%)
Retinal hemorrhage	8 (21.1%)

LogMAR, logarithm of the minimum angle of resolution; BCVA, best-corrected visual acuity; IOP, intraocular pressure; SUN, Standardization of Uveitis Nomenclature; RPE, retinal pigment epithelium

**Table 3:** Origin and involvement patterns of vitreoretinal lymphomas.

	Count/Mean ± Standard Deviation
Primary origin of the lymphomas	
Eye	28 (73.7%)
CNS involvement during follow-up	12 (42.9%)
MRI at routine checkup	9 (75.0%)
Neurological symptoms	2 (16.7%)
Visual-field defects	1 (8.3%)
Brain	10 (26.3%)

CNS, central nervous system; MRI, magnetic resonance imaging.

**Table 4:** Comparisons of the diagnostic test results.

	Count/Mean ± Standard Deviation
Vitreous cytology	38 (100.0%)
Unsatisfactory specimen	11 (28.9%)
Satisfactory specimen	27 (71.1%)
Positive	12 (44.4%)
Negative	15 (55.6%)
Interleukin analysis	28 (73.7%)
IL-10/IL-6 ratio > 1	23 (82.1%)
IL-10 > 50 pg/mL	25 (89.3%)
IGH/IGK gene clonality assay	30 (78.9%)
IGH-positive	18 (60.0%)
IGK-positive	19 (63.3%)
IGH- or IGK-positive	25 (83.3%)

IL, interleukin; IGH, immunoglobulin heavy chain; IGK, immunoglobulin kappa light chain.

**Table 5:** Effect of steroid pretreatment on the diagnostic tests for vitreoretinal lymphomas.

	Steroid Pretreatment (*n* = 12)	No Steroid Pretreatment (*n* = 26)	*p*-Value
	Number/Mean ± Standard Deviation	Number/Mean ± Standard Deviation	
Vitreous cytology	12 (100.0%)	26 (100.0%)	
Unsatisfactory specimen	6 (50.0%)	5 (19.2%)	0.068 *
Satisfactory specimen	6 (50.0%)	21 (80.8%)	
Positive	0 (0.0%)	12 (57.1%)	0.020 *
Negative	6 (100.0%)	9 (42.9%)	
Interleukin analysis	9 (75.0%)	19 (73.1%)	
IL-10/IL-6 ratio > 1	8 (88.9%)	15 (78.9%)	0.999 †
IL-10 > 50 pg/mL	9 (100.0%)	16 (84.2%)	0.530 †
IGH/IGK gene clonality assay	11 (91.7%)	19 (73.1%)	
IGH-positive	8 (72.7%)	10 (52.6%)	0.442 †
IGK-positive	7 (63.6%)	12 (63.2%)	0.999 †
IGH- or IGK-positive	9 (81.8%)	16 (84.2%)	0.999 †

IL, interleukin; IGH, immunoglobulin heavy chain; IGK, immunoglobulin kappa light chain. * Fisher’s exact test; † Chi-square test.

## Data Availability

The datasets generated during and/or analyzed during the current study are available from the corresponding author upon reasonable request.
